# Fatal case of poisoning with a new cathinone derivative: α-propylaminopentiophenone (N-PP)

**DOI:** 10.1007/s11419-018-0417-x

**Published:** 2018-04-27

**Authors:** Milena Majchrzak, Rafał Celiński, Teresa Kowalska, Mieczysław Sajewicz

**Affiliations:** 1 Department of General Chemistry and Chromatography, University of Silesia, 9 Szkolna Street, 40-006 Katowice, Poland; 2 Toxicological Laboratory ToxLab, 6 Kossutha Street, 40-844 Katowice, Poland

**Keywords:** Fatal poisoning, Postmortem specimens, Synthetic cathinones, α-Propylaminopentiophenone, N-PP, LC–MS/MS

## Abstract

**Purpose:**

Similar to synthetic cannabinoids, synthetic cathinone derivatives are the most popular compounds among novel psychoactive substances. Along with a growing number of new cathinones, the number of consumers wishing to enrich their experience with these compounds is also growing, and the same can be said about the growing numbers of poisonings. The reason for overdosing is a lack of consumer awareness regarding composition of the product, with which they experiment, and even more, regarding concentration of psychoactive substances contained in the taken product. In this paper, we report a case of the purposeful intake of a high dose of powder containing a novel cathinone derivative, α-propylaminopentiophenone, which resulted in the deadly poisoning of a woman.

**Methods:**

Aiming to identify this psychoactive substance causing the fatality, the postmortem specimens collected from the autopsy was analyzed by means of high-performance liquid chromatography coupled with mass spectrometry, and the analysis of a powder material found with the victim was additionally analyzed by means of gas chromatography with mass spectrometric detection.

**Results:**

In the course of analysis performed on the specimens originating from autopsy (blood, eyeball fluid, liver, kidney and brain), high concentrations of α-propylaminopentiophenone were established, which was responsible for the death of a young woman. The same psychoactive compound was also identified in the powder material.

**Conclusions:**

To the best of the authors' knowledge, this is the first case reported in the literature on fatal poisoning with α-propyloaminopentiophenone.

## Introduction

Although the history of designer drugs is relatively new (as it only began in the 1990s of the past century), popularity thereof grew rapidly, owing to a fast growing circle of their recipients [1, 2]. In Europe, designer drugs became popular in the second half of the 1990s; they were first imported from New Zealand in the form of tablets known as “party pills” and containing piperazine derivatives [2]. The first stores selling designer drugs emerged in Holland, Great Britain and Germany, and the first Polish store was opened in 2008, in the city of Łódź. The designer drug market is growing with an astonishing speed, with new drug derivatives continuously popping up, whereas identification thereof and risk estimation cannot keep up.

Novel psychoactive substances (NPS) pose a huge problem for legal and clinical toxicologists. New substances rapidly emerge on the market, and great similarity among the compounds belonging to the same chemical group is an additional analytical difficulty. The methods for rapid screening of biological material for designer drugs have not been elaborated with a sufficient success to cover a wide enough spectrum of compounds belonging to different chemical groups. Consequently, a chance to identify psychoactive compounds with patients turning up in hospital wards with a suspicion of poisoning is quite low. The immunochemical tests are limited to selected groups of compounds only, but they are not sensitive and specific enough to identify NPS selectively, nor enable identification of compounds belonging to all NPS groups simultaneously [3]. Legal toxicologists working in forensics laboratories identify novel psychoactive substances using highly specific analytical tools such as high-performance liquid chromatography–mass spectrometry (HPLC–MS) and gas chromatography–mass spectrometry (GC–MS), which enable detailed analysis in a wide range of NPS compounds [4]. Moreover, it is noteworthy that isolation of psychoactive substances from the biological matrix is a very difficult practical task, which generates the need to elaborate the optimal extraction conditions, as extraction is the key step in toxicological analysis of biological specimens.

**Fig. 1:**
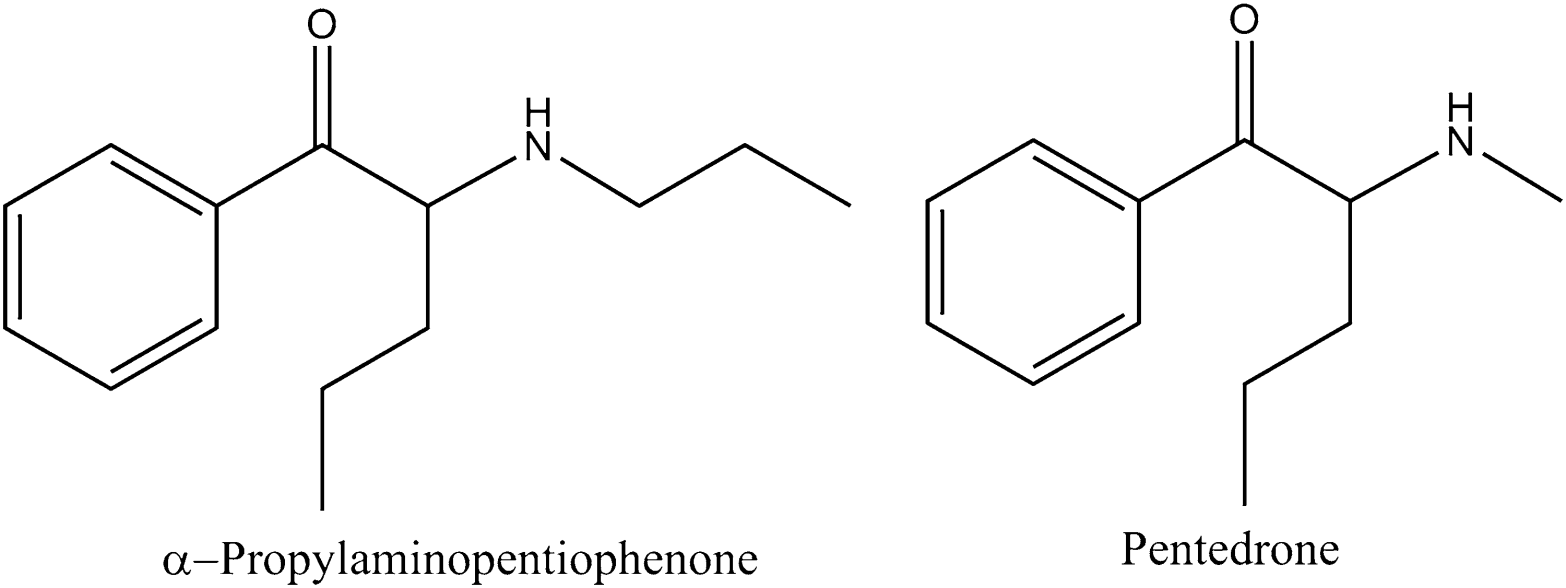
Structures of α-propylaminopentiophenone and pentedrone (internal standard)

Currently, one of the most popular and also most numerous groups among designer drugs are the cathinone derivatives. Each year, much reference information about synthetic cathinones, which dominate the global pseudodrug market, appears in the literature [5–7]. Unawareness of consumers regarding composition of these products, and even more importantly, regarding the concentration of a psychoactive component in the administered dose is the most frequent cause of poisonings, fatalities included. The most recent announcements on fatal overdosing of cathinone derivatives referred to α-pyrrolidinobutiophenone (α-PBP), α-pyrrolidinovalerophenone (α-PVP), 4-methoxy-PV8 (4-methoxy-α-pyrrolidinoheptanophenone), 4-methoxy-PV9 (4-methoxy-α-pyrrolidinooctanophenone) and α-pyrrolidinoctanophenone (PV9) [8–11]. In this study, we report on fatal poisoning with a novel psychoactive compound from the cathinone group, namely α-propyloaminopentiophenone (N-PP) (Fig. 1) and to our best knowledge, this will be the first such case reported in the literature.

## Case history

According to her partner, the 29-year-old woman suffered from depression and alcoholism. She was treated in the past in a hospital for being nervously and mentally ill, and failed a suicide attempt in 2014. At some point on New Year’s Eve 2014, she fought with her partner and left her home. At about 12:30 a.m., the woman returned home under the influence of alcohol. As her partner testified, the woman sat at the table, pulled a bag of white powder out of her pocket, took some of its contents on a flat tea spoon, put it in her mouth, swallowed it, and then repeated with the same portion of the powder again. Then the woman got up and went to the bathroom to take a bath. After a short while, her partner heard her crying for help. She claimed not to feel her legs and was unable to walk on her own. After entering the bathroom, the man saw her in convulsions, and then she lost consciousness and fell to the floor. He alerted the medical emergency service and resuscitated her himself. Further resuscitation activities were carried out first by the police officers and then by the ambulance team. At 02:40 a.m., after a few minutes of resuscitative action, the doctor declared the woman dead. Autopsy revealed cerebral edema, pulmonary edema and passive congestion of internal organs, and it was concluded that acute respiratory distress was the direct cause of death. During the course of examination of the apartment where the death occurred, lines of white powder and blister packs with the drugs Sevredol, MST Continus, haloperidol, Controloc 20 and amitriptyline were secured.

## Materials and methods

### Chemicals

All reagents used in this study were of high purity grade (HPLC and MS). Acetonitrile, 35% ammonia solution, ammonium formate, ethyl acetate, formic acid, methanol, and water Chromasolv were purchased from Sigma-Aldrich (Saint Louis, MO, USA). Samples of the α-propylaminopentiophenone and pentedrone reference standards were purchased from Cayman Chemical (Ann Arbor, MI, USA). The immunoenzymatic ELISA Forensic Drug Test Kits were purchased from Neogen (Lexington, KY, USA).

### Extraction procedure with biological specimens

Biological specimens preserved for the investigations consisted of blood, eyeball fluid, and cuttings from liver, kidney, and brain. The preliminary screening of blood was carried out with use of the immunoenzymatic ELISA technique and it targeted the presence of the compounds from the group of synthetic cathinones, including methcathinone, and other drugs such as amphetamine, tricyclic antidepressants, Δ^9^-tetrahydrocannabinol and benzodiazepines. For the testing, 50-µL blood specimens were diluted with 200 µL buffer provided in the reagent kit of the ELISA test.

Active substances contained in the biological specimens were isolated before HPLC–MS analyses by means of liquid-liquid extraction. To a 2-mL volume of blood or eyeball fluid, 2 mL 15% ammonia (pH 12) and 4 mL acetonitrile containing pentedrone as internal standard (IS) (Fig. 1) were added, and the mixtures were ultrasonicated for 60 min. Then, in each test tube, 1.0 mL each of the mixture was placed (maximum seven tubes), to which different amounts of the target compound and 1.0 mL of ethyl acetate were added. Finally, the test tubes were shaken for 2 h and centrifuged, and the supernatant layer (0.8 mL) was collected and evaporated to dryness in a stream of nitrogen at ambient temperature. The dry residues were dissolved in 1 mL methanol and the samples were transferred to the glass inserts placed in the vials and they were ready for the analyses.

For the cuttings of internal organs (liver, kidney, and brain), each 5-g sample was minced and homogenized and then extracted according to the protocol, using the following reagent volumes: 10 mL 15% ammonia (pH 12), 10 mL acetonitrile, 20 mL ethyl acetate, and 2 mL methanol.

### Extraction procedure with the powder found at the scene

A 10-mg sample of the white powder was dissolved in 1 mL of acetonitrile/methanol (50:50, v/v), ultrasonicated for 10 min and then centrifuged. The 10-μL aliquot of the obtained sample extract was diluted by adding 990 μL acetonitrile/methanol (20:80, v/v). This diluted sample extract was transferred to the glass insert and vial, and then analyzed by means of HPLC–MS or GC–MS.

### HPLC–MS

The qualitative and quantitative HPLC analysis was carried out using a UHPLC Dionex Ultimate 3000 liquid chromatograph (Dionex, Sunnyvale, CA, USA) connected to a TSQ Endura mass spectrometer (Thermo Scientific, Waltham, MA, USA). The chromatograph was equipped with an RP-MS Accucore column (100 × 2.1 mm; Thermo Scientific). Mobile phase was composed of solvent A (0.02 M aqueous solution of formic acid and 0.05 M aqueous solution of ammonium formate) and solvent B (10% solvent A and 90% acetonitrile). The analyses were carried out in the gradient mode, using the following gradient: 0–2 min, 95% A + 5% B; 2–30 min, 30% A + 70% B; 30–32 min, 30% A + 70% B; 32–40 min, the volume ratio was returning to the initial one (95% A + 5% B). The mobile phase flow rate was 100 µL/min. Samples were ionized in the electrospray ionization (ESI) mode with positive ionization, and the ion monitoring range was *m*/*z* 50 to 500.  The temperature of the ion source was 250 °C; nitrogen was the carrier gas and helium was the ionization gas. For the selected reaction monitoring (SRM) mode, the ion transitions were *m*/*z* 220.1→ 202.1 and *m*/*z* 192.1→ 174.1, for α-propylaminopentiophenone and IS, respectively. The results obtained were processed with use of the Xcalibur v. 4.0. program.

### GC–MS

The GC–MS analysis was carried out with use of the Trace 1310 model gas chromatograph (Thermo Scientific) equipped with the Rxi^®^-5Sil MS column (15 m × 0.25 mm, film thickness 0.2 µm; Restek, Bellefonte, PA, USA) coupled with the TSQ 3000 Thermo Scientific mass spectrometer (Thermo Scientific). The employed working conditions: the injector temperature, 260 °C; the oven temperature, 100 °C for 2 min, then the temperature rise to 260 °C at the rate of 20 °C/min; the carrier gas, helium, its flow rate, 1.2 mL/min; the temperature of the MS source, 250 °C; the injection volume, 1 μL. The obtained mass spectra in the positive electron ionization (EI) mode were compared with the spectra from the Cayman Chemical EI mass spectra library (2017) (Cayman Chemical).

### Standard addition method

In order to quantify α-propylaminopentiophenone in the autopsy specimens, the standard addition method was employed. This method can overcome the matrix effects and recovery rate differences [9, 10]. Moreover, the method requires no blank human specimens that are negative for a target compound. It is true that the collection of blank human tissue samples is problematic for ethical reasons. On the other hand, that method can be used by entities that do not have the possibility to acquire blank biological specimens. To obtain a single concentration value, a calibration curve established with the seven concentrations equal to 20, 50, 100, 200, 500, 1000 and 5000 ng/mL of the target compound needs to be constructed. The different concentrations of target compound and fixed concentrations of IS were added to each portion of the same matrix as described before. The minus concentration where the straight standard addition calibration curve intersected with the horizontal x-axis of target compound concentration showed the existing concentration of the target compound in the matrix.

### Matrix effects and recovery rates

First, to determine matrix effects and recovery rates in all analyzed fluids and tissues, a concentration of target compound in each matrix was measured by the standard addition method. Then, we prepared a reference standard α-propylaminopentiophenone methanol solution at the concentration, which could be expected in the finally reconstituted methanol solution assuming that the extraction recovery of the target compound was 100% for each matrix, which corresponded to the below methanol solution C. Each matrix was prepared in the procedure described before with addition of IS in the early step. At the final step, where 1 mL (for fluid) and 2 mL (for solid tissues) of pure methanol was added and mixed to reconstitute the sample extract residue after evaporation. In addition, 1 or 2 mL of the methanol solutions containing the 100% recovery final concentrations of α-propylaminopentiophenone was added to another sample extract residue and mixed. Therefore, there are three types of methanol solution to be injected to HPLC–MS. The one obtained from a matrix after reconstitution of the sample extract residue with pure methanol was denoted as B (by means of peak area); the one obtained after reconstitution with methanol containing the 100% recovery concentration of the target compound for the same sample extract was denoted as A (by means of peak area). The methanol solution without any reconstitution, but only with the above 100% recovery concentration of the target compound was described as C (neat sample). The matrix effect and recovery rate were calculated as follows. Matrix effect (%) = [(A − B)/C] × 100. Recovery rate (%) = [B/(A − B)] × 100.

## Results and discussion

### GC–MS mass spectrum obtained from the powder

**Fig. 2:**
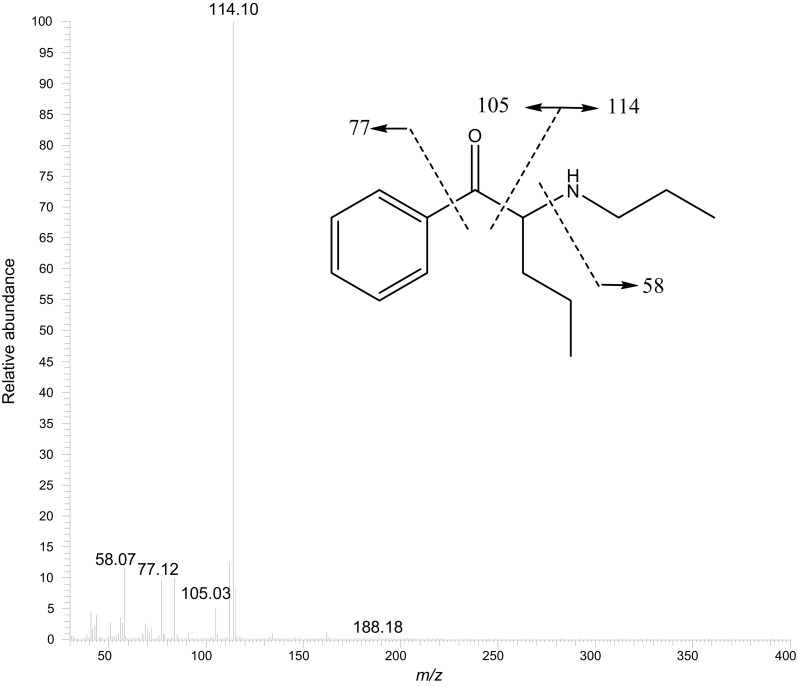
Electron ionization mass spectrum of α-propylaminopentiophenone obtained by gas chromatography–mass spectrometry from the powder

The EI-MS spectrum originating from the GC–MS analysis was checked with use of the mass spectrum included in the Cayman Chemical Library, which suggested α-propylaminopentiophenone as the major ingredient of the powder. Then, the spectrum and retention time of the ingredient were confirmed to be identical with those of reference standard of α-propylaminopentiophenone. The peak at *m*/*z* 114.11 appeared as the base peak, and the peaks at *m*/*z* 105.03, 77.12 and 58.07 appeared as fragmentation ions (Fig. 2). The total ion current chromatogram showed that the peak area of α-propylaminopentiophenone constituted 90% of the sum of peak areas, suggesting its purity at about 90%.

### Electrospray ionization product ion spectrum obtained from the powder

**Fig. 3:**
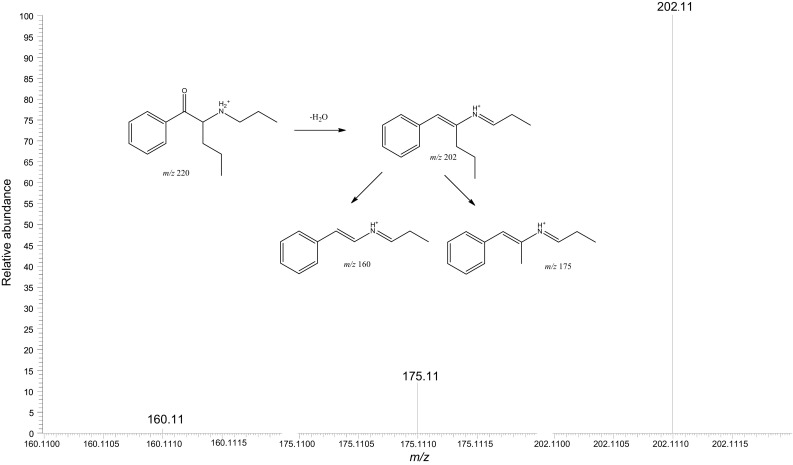
Product ion spectrum of α-propylaminopentiophenone obtained from the powder sample found at the scene together with the proposed fragmentation pattern

The protonated molecular ion [M + H]^+^ at *m*/*z* 220.17, which was obtained from the white powder, underwent further MS/MS fragmentations in the positive ionization mode. The MS^2^ spectrum valid for the ion at *m*/*z* 202.11 suggested elimination of one molecule of water [M + H − H_2_O]^+^. This transition is well recognized and characteristic of the compounds belonging to the cathinone group, which suggests the presence of such compound in the analyzed sample [12]. Other product ions with the *m*/*z* values of 175.11 and 160.11, respectively, corresponded well with the structural fragments of α-propylaminopentiophenone (Fig. 3.)

### The ELISA tests

As a result of screening of biological samples, the blood specimen with use of the immunoenzymatic ELISA test, showed the positive response for the synthetic cathinones. Because of the lack of similar tests with antibodies valid for eyeball fluid, liver, kidney and brain, the ELISA screening tests for the remaining organs could not be performed.

### HPLC–MS analysis of the powder and human specimens

**Fig. 4:**
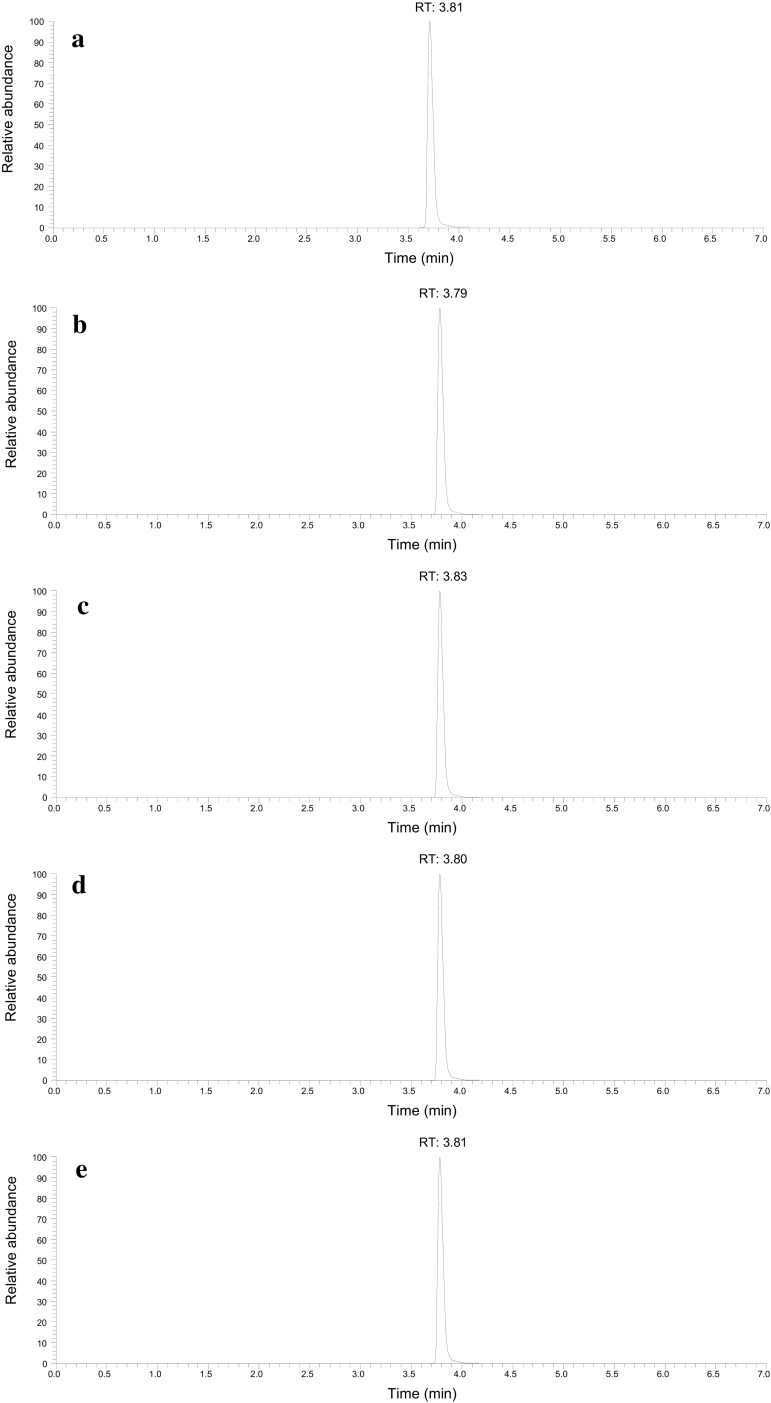
Selected reaction monitoring chromatograms obtained from high-performance liquid chromatography with tandem mass spectrometric detection of α-propylaminopentiophenone for the specimens of **a** blood, **b** eyeball fluid, **c** liver, **d** kidney, and **e** brain

**Fig. 5:**
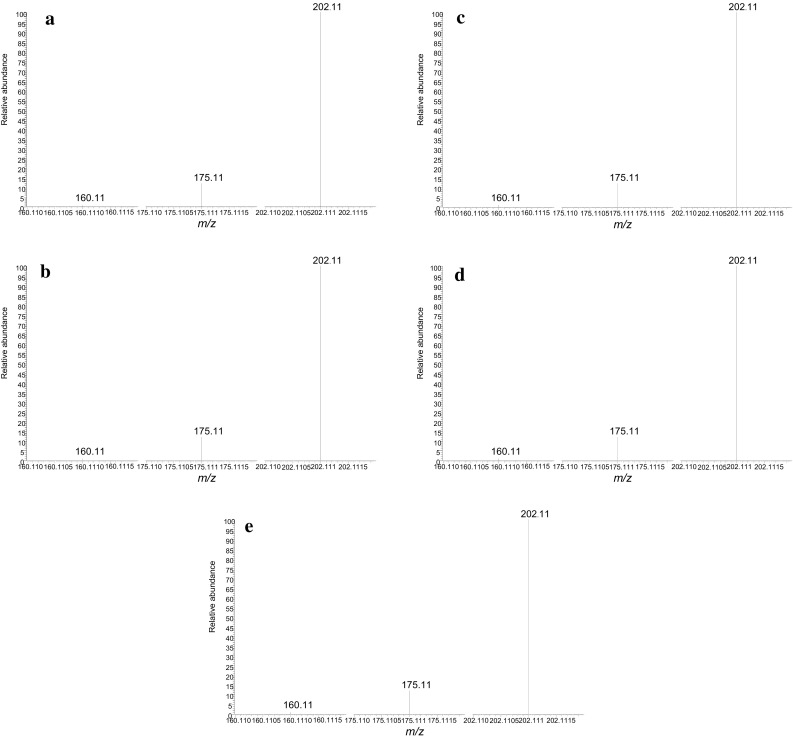
Product ion spectra of α-propylaminopentiophenone for the specimens of **a** blood, **b** eyeball fluid, **c** liver, **d** kidney, and **e** brain

By analysis in the SRM mode of the powder, the α-propylaminopentiophenone reference standard, and the biological specimens, the chromatograms were obtained with the peak at the retention time in the range of 3.79–3.83 min. The SRM chromatograms showed only a single peak of the target compound; no other impurity peaks ever appeared for any specimen (Figs. 4a–e). The ESI product ion spectra showed only the peaks due to α-propylaminopentiophenone for all specimens; no impurity peaks appeared (Fig. 5a–e).

### Quantitative analysis of human samples

The standard addition calibration equations and their correlation coefficients (*r*) for body fluids and solid tissues are shown in Table 1. The limit of detection [LOD, signal-to-noise (*S/N*) ratio = 3] and limit of quantification (LOQ, *S/N* ratio = 10) were calculated. The LOD and LOQ values were 9.0 and 27.2 ng/mL for body fluids and 15.0 and 46.0 ng/g for solid tissues, respectively. Because we did not have blank human specimens, it was not possible to present usual accuracy and precision. However, we evaluated the intraday and interday repeatability of target compound for each matrix. The results are shown in Table 2. The relative standard deviations (RSD) of intraday and interday repeatabilities were 6.4–22% except for interday brain measurement (RSD = 40%). As also shown in Table 2, among five specimens tested, the highest concentration of α-propylaminopentiophenone was observed (5.9–6.0 µg/g) in the liver, while the lowest one was found in the brain (2.1–2.3 µg/g). As described earlier, we have also evaluated the matrix effects and recoveries for each type of biological specimens and the results are shown in Table 3. The matrix’s largest influence was observed in the brain, whereas the highest recovery was in the liver, although all data were within the acceptable range.

**Table 1:** Standard addition calibration equations and correlation coefficients for α-propylaminopentiophenone in human specimens collected at autopsy

Human specimen	Equation	Correlation coefficient (*r*)
Blood	*y* =0.3264 *x *+0.0223	0.998
Eyeball fluid	*y* =0.0997 *x *+0.2684	0.996
Liver	*y* =0.1147 *x *+0.0954	0.997
Kidney	*y* =0.2244 *x *+0.1090	0.995
Brain	*y* =0.8992 *x *+0.2151	0.995

**Table 2:** Evaluated intraday and interday repeatability for determination of α-propylaminopentiophenone in each human specimen

Specimen	Intraday (*n* = 5)		Interday (*n* = 5)	
	Concentration^a^ (µg/mL or g)	Repeatability (% RSD)	Concentration^a^ (µg/mL or g)	Repeatability (% RSD)
Blood	3.2 ± 0.68	21	3.1 ± 0.23	7.4
Eyeball fluid	4.4 ± 0.52	12	4.2 ± 0.90	21
Liver	5.9 ± 0.38	6.4	6.0 ± 0.66	11
Kidney	5.4 ± 0.92	17	5.4 ± 1.20	22
Brain	2.3 ± 0.50	22	2.1 ± 0.85	40

*RSD* relative standard deviation

^a^Data were expressed as mean ± standard deviation (SD)

**Table 3:** Matrix effects and recovery rates for determination of α-propylaminopentiophenone in each human specimen

Specimen	Matrix effect ± SD (%)	Recovery ± SD (%)
Blood	74.1 ± 4.6	75.5 ± 5.0
Eyeball fluid	70.3 ± 5.8	71.3 ± 3.6
Liver	84.8 ± 3.2	82.6 ± 3.2
Kidney	80.3 ± 4.7	72.8 ± 1.6
Brain	68.8 ± 6.4	64.3 ± 3.4

Data given as mean ± SD (*n* = 3)

From the literature, it is known that the range of concentrations for the cathinone derivatives established in the tissues collected from fatal cases is very wide and it largely depends on the type of the analyzed biological specimens, as well as on additional circumstances preceding the death [8–11, 13]. Concentrations of cathinone derivatives in autopsy blood obtained from cadavers so far reported in the literature have been in the range of 0.082–22 µg/mL [14]. In these cases, when passing away takes place soon after overdosing on a given designer drug, its concentrations in the organs are usually high. When the detoxication attempts (e.g., through gastric lavage or intravenous infusion) are performed prior to the person’s passing away, concentration of a given cathinone in the postmortem blood is lower, which seems quite logical [15]. In the case reported in this reference, prior to death the person underwent an intense detoxication process, so that concentrations of PV9 established in nine different body tissues were in the range from 0.212 to 0.907 µg/g. Concentrations of cathinone derivatives such as 4-methoxy-PV8, PV9 and 4-methoxy-PV9 in the postmortem blood of a young woman who most probably died shortly after a simultaneous intake of all three was 2.69, 0.743 and 0.261 µg/mL, respectively [11]. Ethyl alcohol was additionally found at the 1.52 mg/mL level in the postmortem blood of the deceased woman, which might additionally strengthen toxic action of the cathinone derivatives. Nevertheless, damage of internal organs due to an intake of high amounts of synthetic cathinones which quickly penetrate to the bloodstream is enough to result in passing away, independent of earlier undertaken detoxication attempts.

Concentrations of α-propylaminopentiphenone found in blood, eyeball fluid, liver, kidney and brain of the deceased reported in this study were very high (Table 2) and they clearly explain rapid reaction of the young woman’s organism and her eventual quick death. It happened due to an intake of high quantities (two spoonfuls) of the drug powder (probably ± 10 g), whereas the average portions taken by non-addicts range between 20 and 200 mg powder [16].

## Conclusions

As a result of the postmortem analysis of blood, eyeball fluid, liver, kidney, and brain, high concentrations of α-propylaminopentiophenone in these organs were disclosed. Based on known circumstances of deaths in combination with the analytical data obtained, the young woman seemed susceptible to fatal poisoning by α-propylaminopentiophenone from the cathinone group. To the authors’ best knowledge, this is the first case reporting fatal poisoning with a novel psychoactive compound α-propyloaminopentiophenone.
